# Analysing post-apartheid gender and racial transformation in medical education in a South African province

**DOI:** 10.3402/gha.v6i0.19810

**Published:** 2013-01-24

**Authors:** Taskeen Khan, Leena S. Thomas, Shan Naidoo

**Affiliations:** 1 Gauteng Health Department, Charlotte Maxeke Johannesburg Academic Hospital, Johannesburg, South Africa; 2 School of Public Health, Faculty of Health Sciences, University of the Witwatersrand, Johannesburg, South Africa; 3 Gauteng Health Department, Ekurhuleni, Gauteng, South Africa

**Keywords:** medical education, transformation, race, gender, inequalities

## Abstract

**Introduction:**

In light of global concerns about insufficient numbers of doctors, midwives, and nurses, the World Health Organization (WHO) has identified the scale-up of the production of medical professionals who are competent and responsive to community needs as urgent and necessary. Coincident with this imperative, South African medical schools have also had to consider redressing apartheid-era inequities in access to medical education and changing the racial and gender profile of medical graduates to be representative of the population. In this article, we explore progress and challenges with regard to transformation, defined as intentional and planned changes aimed at addressing historical disadvantages, in the Gauteng Province of South Africa.

**Methods:**

A cross-sectional, descriptive analysis was conducted using data on medical school admissions and graduations from the Health and Education Departments for the period 1999–2011. Admission and graduation statistics of 1999, 2005, 2008, and 2011 were analysed according to race and gender.

**Results:**

The results show that there has been progress in transforming the race and gender composition of medical students and graduates, in line with the transformation strategies of the South African government. In 1999, black African enrolments and graduates were conspicuously low in two of the three medical schools in the Gauteng province. By 2011, an almost six-fold increase in black African student enrolments was seen in one medical school that was previously designated as a white institution. In contrast, at the historically black medical school, whites only represented 0.40% of enrolments in 1999 and 7.4% in 2011. Since 1999, the number and proportion of female medical enrolments and graduates has also increased substantially.

**Conclusion:**

While there has been progress with redressing historical disparities and inequities in terms of race and gender, further efforts are needed to ensure that student intakes and graduations are in line with the South African population profile.

Despite major medical advances and breakthroughs during the 20th century, global inequities in health and access to care still persist (1, 2). The World Health Organization (WHO) estimated that more than a billion people worldwide lack access to quality healthcare, partly due to the major shortages, skill imbalances, and the inequitable distribution of doctors, nurses, and midwives. It is further estimated that 2.4 million more doctors, nurses, and midwives are needed globally to address current and future health needs (3). However, not enough health professionals are being educated, particularly in Africa where they are needed the most, exacerbated by insufficient collaboration between the health and education sectors (2). For this reason, the WHO has identified the scale-up of medical education as being both necessary and urgent. However, even well-educated graduates may not have the necessary or relevant skills or the content of their education could be unrelated to the epidemiological profile and needs of the populations they serve. In addition, the burden of disease, teaching patterns, and retention of personnel in the public sector play a role in producing health professionals relevant to a country's needs (3). Thus, health workers should graduate with appropriate competencies, thereby meeting population health and health service needs (3).

In many countries, medical education reforms in higher education institutions are often part of broader social transformation efforts. In this article, transformation refers to “an intentional social, political, and intellectual project of planned change aimed at addressing historical disadvantages, inequities, and serious structural dysfunctions” (4). For example, countries in eastern and central Europe, Japan, Brazil, India, Australia, Cameroon, and the United States of America (USA) have introduced various medical education reforms in the past two decades. The aim of these reforms is to address gross disparities and inequities among social classes and minority groups (Brazil, India, Australia, and USA) and/or to overcome histories of repressive regimes (eastern and central Europe) (5). In South Africa, medical schools have also had to consider redressing apartheid-era inequities in access to medical education and changing the racial and gender profile of medical graduates to be representative of the population. It is beyond the scope of this article to address all aspects of transformation. Instead, we explore whether transformation has been achieved in post-apartheid South Africa and if the gender and racial profile of medical students and graduates are in line with the national demographic profile of the country. This article may hold lessons for the transformation of medical education in other societies where disparities or inequalities are still common.

## Background

### South African context

**Fig. 1:**
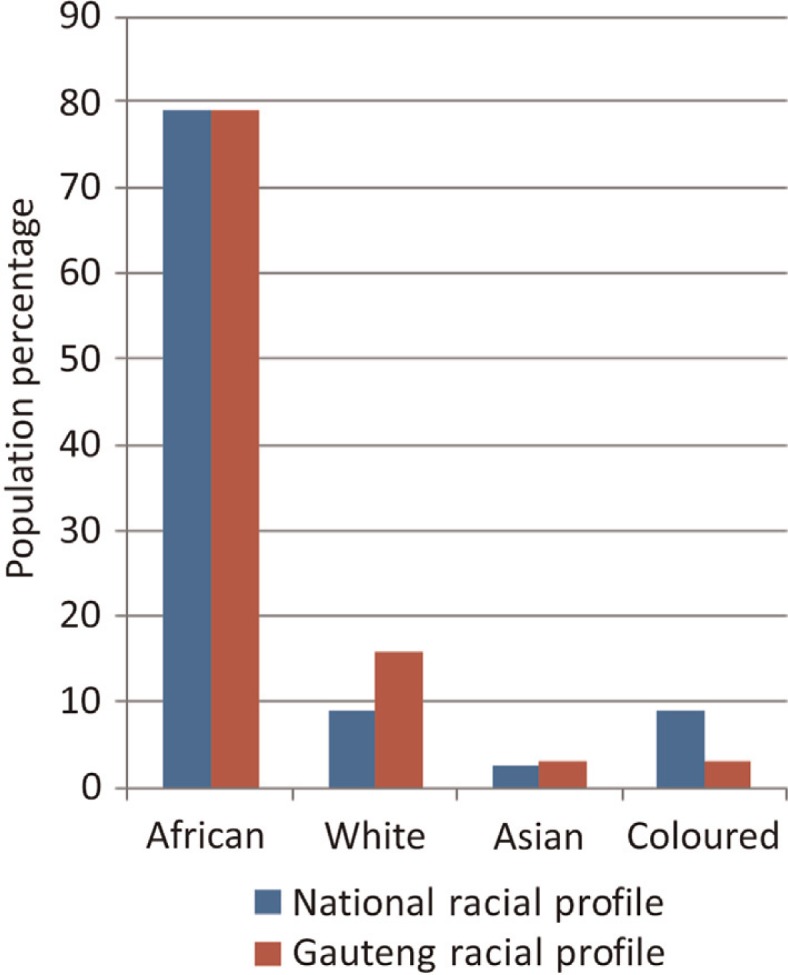
National and Provincial racial profiles (2011).

South Africa is home to 51 million people. The ‘racial’ composition of the South African population, a social construct, comprises black Africans (79%), Asians (mainly Indian and a very few Chinese) (2.5%), coloureds (9%), and whites (9%). In the Gauteng province, the focus of this study, there is a higher proportion of whites, compared to the national profile (Fig. 1) (6).

Discrimination and exclusion policies on the basis of race and gender, startling income inequalities, and violence have all formed part of South Africa's troubled past and have affected all social, political, and economic aspects of South African society (7). Similarly, population health and healthcare challenges have been exacerbated by the legacies of colonialism and apartheid, which have also influenced medical education.

Although black Africans are in the majority, during apartheid they were denied or had limited access to tertiary education. Consequently, they were not represented adequately amongst medical professionals during apartheid. Such discriminatory and exclusive education policies created disparities and inequalities in two ways: first, by ensuring resources were allocated in accordance with the apartheid ideology (unequally based on race), and second, by treating people not classified as white in an inferior and discriminatory manner (8). The distorted state of the medical fraternity in South Africa prior to 1994 is illustrated by the Medical Association of South Africa's submission to the Truth and Reconciliation Commission, which described the organization as *white, male, elitist, educated and professional* (9).

## Overview of reforms in medical education

Currently there are eight universities in South Africa that train medical doctors (10). Under apartheid, universities with medical schools were designated to train medical professionals of certain race categories. Under apartheid, only three of the medical schools in the country were allowed to train black African students (11). Medunsa (now known as the Health Science Faculty of the University of Limpopo) and Unitra (now known as Walter Sisulu University) catered exclusively for African students, while Natal (now known as the University of Kwazulu Natal) serviced both Indian and African students (11).

Until the late 1980s, apartheid policies dictated that the five historically white universities had stricter admission criteria for black African students and only very few were admitted. During the 1980s, some of the more liberal universities attempted to admit more African students into medicine. However, due to inferior secondary schooling and stringent admission criteria, black African students remained very few in numbers (12). After 1994, the new democratic government introduced profound reform policies and transformation in the health and education sectors to redress historical inequities (9). The National Commission on Higher Education was established in 1995 to advise the minister of education on the restructuring of higher education so as to contribute toward reconstruction and development in South Africa (8). This led to a number of transformation policies such as the White Paper on Higher Education (8). The latter set out to restructure higher education into a single, nationally coordinated system that would redress the inequities created by apartheid and meet the needs of a new South Africa with fundamentally changed economic, social, and political structures (13).

Restructuring of medical schools in terms of gender and race enrolments as well as curriculum reforms were seen as necessary steps in transforming human resources for health in South Africa. A number of reform measures have been introduced in medical schools to overcome historical disparities, inequalities, and inequities in medical education. Discrimination and exclusion based on race is outlawed in the South African constitution and all medical schools have adjusted their admission policies to admit students regardless of race or gender (10). There is also a significant move toward increasing the number of black African medical students, particularly in historically ‘white’ institutions. The same applies to the number of female medical student admissions, which has increased considerably. In addition, the curriculum of medical education has been revisited, while problem-based learning now forms a major strategy in some universities. Other initiatives include the development of community education sites and the introduction of a graduate entry medical programme at one university, which admits individuals who have successfully completed an undergraduate degree, thus offering many students who may not have been eligible straight after school an alternative entry route to medical training.

While there is ample information on the history of race in medical education, there is less on the history of gender in the medical profession. Past studies have illustrated the predominance of males in the profession showing notable historical differences in intake, but in recent years an increasing trend of female enrolments is seen (14). In this article, we explore this further.

## Study setting

Gauteng, one of the nine provinces in South Africa, has the highest gross domestic product (GDP) in the country and is known as the ‘economic hub’ of South Africa. It hosts three medical schools, two of which historically trained white medical students, and the third was exclusively for black African students. These three schools account for almost half of all medical student enrolments and graduates in the country (15). In light of this and because of practical considerations, the Gauteng Province is the focus of this analysis.

## Methods

The focus of the study was the period from 1999 to 2011, and 1999 was used as the base year. A key assumption of the study was that by 1999, that is, 5 years after the onset of democracy, admission policies would have changed at medical schools and graduation statistics would begin to reflect this change. Using 1999 data as the baseline, consecutive 3-year follow-up data were used for 2005, 2008, and 2011 in an attempt to determine trends over the 12-year period.

A cross-sectional, descriptive analysis was conducted, using enrolment and graduate data for the three medical schools in Gauteng. The data were sourced from the Gauteng Department of Health (GDH) for the period 2008–2011. The GDH data were only available from 2007. For this reason, data for 1999 and 2005 were sourced from the Department of Education (DoE), captured in its Higher Education Management Information System (HEMIS). Race is categorised into black African, Asian, coloured, and white enrolments and graduates, while gender is reflected as female (F) or male (M).

The three medical schools are referred to as A, B, and C, respectively. During the years of apartheid, medical school A catered to the training of African students, medical school B used to train Afrikaans-speaking white students, and medical school C was mainly for English-speaking white students.

The collected data were entered and analysed in Microsoft Office Excel 2007.

Tests of statistical significance were carried out in Epi Info (version 3.5.3) and a Chi-square test was used to determine statistical significance.

## Results

**Fig. 2:**
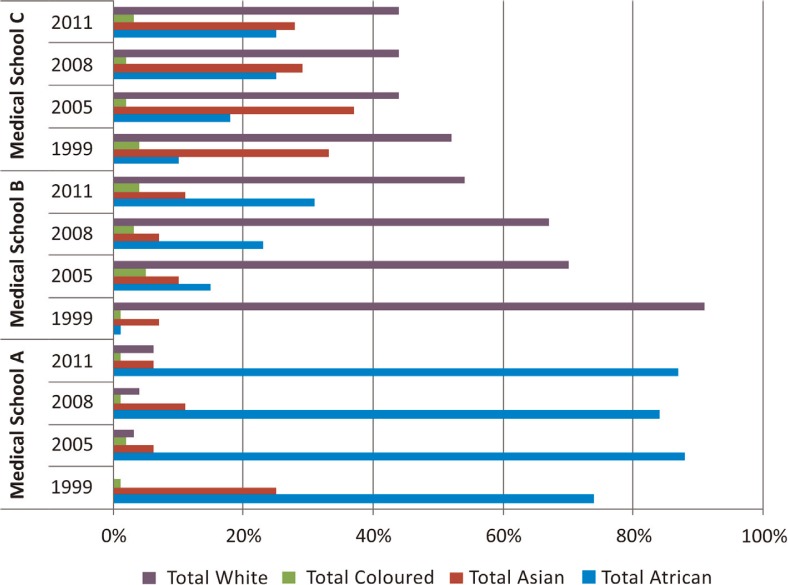
Medical graduates by race and medical school.

Table 1 shows figures for 1999, 2005, 2008, and 2011 for medical enrolments and graduates. In 1999, the numbers of black African enrolments and graduates were low at medical schools B and C. These numbers have subsequently increased. In 1999, black African students at school B represented 9.7% of all enrolments; in 2011, this proportion had increased to 30.1%. Similarly at school C, black African students represented only 5.8% of enrolments in 1999, which increased to 44% in 2011. At school A, white medical students represented a mere 0.40% of enrolments in 1999, whereas they comprised 7.4% in 2011. A similar pattern can be observed regarding the numbers and proportions of medical graduates among the different race groups, although these numbers are notably smaller. Figure 2 is a graphical representation of percentages of medical graduates, stratified by race and medical school, for the years 1999, 2005, 2008, and 2011. The graph shows that in 1999, medical school A had 74% black African graduates, with 0% white and coloured graduates. In 2011, 87% were African, 1% coloured, and 6% white. During the same period, the proportion of Asian graduates at school A decreased drastically from 25% in 1999 to 6% in 2011. At medical school B, an almost opposite trend is seen: in 1999, a mere 1% of graduates were black African compared to 91% white. In 2011, black Africans accounted for 31% of medical graduates and whites accounted for 54% at school B. This equates to an almost 50% reduction of white graduates at school B. The number of coloured graduates rose significantly from 1% in 1999 to 4% in 2011, with Asian graduates also increasing in number. At medical school C, black African graduates in 1999 constituted 10% and white graduates 52% of all graduates. In 2011, the proportion of black African graduates has grown to 25%, while white graduates stood at 44%. Asian graduates dropped from 33% in 1999 to 28% in 2011, percentage coloured graduates also dropped slightly.

**Table 1:** MBChB enrolments and graduations at individual Gauteng medical schools by race and gender

Institution		Year	African		Total	Asian		Total	Coloured		Total	White		Total	Grand Total
			
			F	M		F	M		F	M		F	M		
Medical school A	Enr	1999*	545	783	1328	121	242	363	8	13	21	2	5	7	1719
	Enr	2005*	536	702	1238	42	52	94	9	7	16	21	30	51	1399
	Enr	2008	442	569	1011	31	25	56	5	3	8	25	35	60	1135
	Enr	2011	520	571	1091	31	29	60	3	6	9	41	52	93	1253
Total A enrolments			2043	2625	4668	225	348	573	25	29	54	89	122	211	5506
	Grad	1999*	77	148	225	23	54	77	2	2	4	0	0	0	306
	Grad	2005*	109	151	260	5	14	19	2	3	5	4	6	10	294
	Grad	2008	74	85	159	9	11	20	1	1	2	3	5	8	189
	Grad	2011	67	79	146	4	7	11	1		1	6	4	10	168
Total A graduates			327	463	790	41	86	127	6	6	12	13	15	28	957
Medical school B	Enr	1999*	65	57	122	41	38	79	10	20	30	522	500	1022	1253
	Enr	2005*	212	117	329	62	53	115	34	17	51	468	322	790	1285
	Enr	2008	252	140	392	54	41	95	27	15	42	487	268	755	1284
	Enr	2011	270	131	401	49	33	82	22	22	44	528	276	804	1331
Total B enrolments			799	445	1244	206	165	371	93	74	167	2005	1366	3371	5153
	Grad	1999*	1	1	2	4	9	13	0	3	3	86	93	179	197
	Grad	2005*	18	12	30	11	8	19	5	4	9	79	60	139	197
	Grad	2008	34	13	47	4	11	15	4	1	5	72	61	133	200
	Grad	2011	42	22	64	15	7	22	5	3	8	71	43	114	208
Total B graduates			95	48	143	34	35	69	14	11	25	308	257	565	802
Medical school C	Enr	1999*	74	84	158	286	236	522	12	17	29	332	236	568	1277
	Enr	2005*	204	239	443	226	133	359	17	7	24	294	133	427	1253
	Enr	2008	293	233	526	168	91	259	28	10	38	247	167	414	1237
	Enr	2011	382	199	581	133	90	223	53	25	78	268	171	439	1321
Total C enrolments			953	755	1708	813	550	1363	110	59	169	1141	707	1848	5088
	Grad	1999*	11	11	22	47	25	72	3	6	9	64	49	113	216
	Grad	2005*	16	29	45	56	35	91	3	1	4	70	37	107	247
	Grad	2008	23	24	47	36	19	55	3		3	54	30	84	189
	Grad	2011	31	13	44	35	13	48	2	3	5	44	33	77	174
Total C graduates			81	77	158	174	92	266	11	10	21	232	149	381	826

* Data obtained from Department of Education, HEMIS, 2007.

The other important dynamic analysed was the gender differences in admissions and graduates at the three medical schools for 1999, 2005, 2008, and 2011. All three medical schools have shown rising trends in the proportions of both female enrolments and graduates. In 2011, medical school A was the only institution where less than half of all enrolments and graduates were female. In medical schools B and C, more than half of enrolments and graduates were female since 1999, a large proportion of whom were white.

The differences by ‘race’ totals in the different institutions were statistically significant (*p*<0.0000). These values were the same for both enrolments and graduates.

## Discussion

The results suggest that Gauteng medical schools are implementing government's transformation strategies. This progress is reflected in changing race- and gender-numbers and proportions of medical student enrolments and medical graduates over the 12-year period. These findings are consistent with the published literature (11, 13).

Medical school B has been particularly active in revising its enrolment policy and aligning itself better with national and provincial population demographics. Medical school A is also not fully representative of the national population but is still more aligned than the other two medical schools though their coloured student intake is still low. In order for the medical schools to be representative of national and provincial demographics (Fig. 1), medical schools B and C – with just 31% and 25% of their graduates being black African in 2011 – need to increase these graduates. Similarly, medical school A needs to increase their white graduates. Coloured student graduates comprise one-fourth of their proportion reflected nationally and need to be increased across the three medical schools. This could be due to the low proportion of coloureds in Gauteng (3%) compared to the national figure (9%) and to the province of the Western Cape where coloureds constitute more than 50% of the population (16). Studies from the Western Cape illustrate that despite higher proportions of coloureds in the province, only a very small proportion of them are accessing undergraduate medical programmes (17). As a marginalised community, it could be argued that there needs to be a more concerted effort from all the stakeholders involved in selecting, supporting, and mentoring coloured students in health science training in Gauteng and other provinces.

The ‘brain drain’ affecting health systems in sub-Saharan Africa has been well documented. In particular, South African doctors are much in demand internationally and the country is one of those severely affected by the ‘brain drain’ (18, 19). A study found that 45% of students who had graduated from medical school C since 1975 were working abroad (20). Price and Weiner also examined graduate data from 1960 to 1994 and found that a large proportion of medical graduates in this period had also worked mainly in the private sector in South Africa, with only their initial work years spent in the public health sector in South Africa (21). The majority of these graduates were found to be white. While the role of race in emigration and public sector retention can only be speculated here, there is no guarantee that if we train more black African students, or more students from disadvantaged backgrounds, that they will not succumb to the attractions of emigration and the private sector. However, the WHO does lean toward supporting such initiatives (3). Nonetheless, other studies conducted on this topic have been inconclusive and the advice is to look at country-specific innovations to deal with public sector staff attrition and retention (22).

Female students (63%) dominate the graduate figures in the Gauteng province and all three medical schools have exceeded the national proportion of females in the population. There has been some discussion in the literature regarding the feminisation of the profession and the implications it poses, particularly for postgraduate surgical studies (14). In addition, females are still viewed as child carers in the traditional society and the medical profession is not adequately resourced to take this into account. South Africa needs to adapt and think of innovative ways to address these challenges (14).

Notwithstanding the importance of context when examining transformation efforts, there are lessons for other countries from this South African case study. A combination of legislation and policies is needed for redressing historical gender and racial inequalities, and to support efforts in curriculum reform and other educational reforms.

At the same time, continuous and systematic monitoring and evaluation of the implementation of the various policies and legislation that intend to facilitate transformation in medical education are needed (8). This could set benchmarks, which in turn could be used to enhance existing policy implementation or inform new policies and/or legislation.

## Limitations

The study has several limitations.

First, the data for the years analysed were limited to the information available from the health and education departments.

Second, as a result of democracy and globalisation, South Africa has experienced an influx of economic and political migrants from other parts of Africa and the rest of the world. After 1994, many of these migrants are now naturalised South Africans, and University admissions do not, and possibly cannot, reflect these nuances. Therefore, a student classified as ‘black African’ may not necessarily be a previously disadvantaged South African individual as they may originally have come from other African countries.

Third, only data from Gauteng institutions were used, which cannot be generalised to the rest of South Africa.

Fourth, enrolments and graduates could not be compared because programme duration varies between 5 and 6 years at the different medical schools.

Finally, the study was limited to an analysis of gender and race dynamics. There was no focus on other aspects of transformation, the perceptions of historically disadvantaged students, or on the impact of curriculum reforms and transformation on population health or service delivery. Further research is needed in these areas.

## Conclusion

There has been encouraging progress on medical student admissions and graduations with respect to gender and race in Gauteng Province, achieved through a combination of enabling legislation, policies, and institutional efforts.

However, further efforts are needed to ensure that student intake and graduations are in line with national and provincial demographic profiles.

## References

[1] Woollard RF (2006). Caring for a common future: medical schools’ social accountability. Med Educ.

[2] Frenk J, Chen L, Bhutta ZA, Cohen J, Crisp N, Evans T (2011). Health professionals for a new century: transforming education to strengthen health systems in an interdependent world. Revista Peruana de Medicina Experimental y Salud Pública.

[3] World Health Organisation (2011). Transformative scale up of health professional education [Internet].

[4] Aina TA (2010). Beyond reforms: the politics of higher education transformation in Africa. Afr Stud Rev.

[5] Maassen P, Cloete N (2006). Global reform trends in higher education. Transform Higher Educ.

[6] Statistics South Africa [Internet] (2011). Pretoria.

[7] Coovadia H, Jewkes R, Barron P, Sanders D, McIntyre D (2009). The health and health system of South Africa: historical roots of current public health challenges. The Lancet.

[8] Reddy T (2004). Higher education and social transformation: a South African case study.

[9] University of Cape Town Truth and reconciliation a process of transformation at UCT health sciences faculty [Internet].

[10] Lehmann U, Andrews G, Sanders D (2000). Change and innovation at South African Medical Schools: an investigation of student demographics, student support and curriculum innovation. Health Systems Trust [Internet].

[11] Burch VC (2007). Medical education in South Africa: assessment practices in a developing country [Internet].

[12] Colborn RP (1995). Affirmative action and academic support: African medical students at the University of Cape Town. Med Educ.

[13] Roberts DRD (2006). Governance in Higher Education in South Africa: a transformation and development perspective [Internet]. The Fourth Pan-Commonwealth Forum on Open Learning (PCF4).

[14] Breier M, Wildschut A (2008). Changing gender profile of medical schools in South Africa. S Afr Med J.

[15] Breier M (2008). The shortage of medical doctors in South Africa [Internet]. Human Sciences Research Council.

[16] Statistics South Africa [Internet]. http://www.statssa.gov.za/census01/census96/HTML/press/Part017.html.

[17] Breier M, Wildschut A Doctors in a divided society: the profession and education of medical practitioners in South Africa.

[18] Muula AS (2005). Is there a solution to the ‘brain drain’ of health professionals and knowledge in Africa?. Croat Med J.

[19] Hagopian A, Thompson MJ, Fordyce M, Johnson KE, Hart LG (2004). The migration of physicians from sub-Saharan Africa to the United States of America: measures of the African brain drain. Hum Res Health.

[20] Weiner R, Mitchell G, Price M (1998). Wits medical graduates: where are they now?. S Afr Med J.

[21] Price M, Weiner R (2005). Where have all the doctors gone? Career choices of Wits medical graduates. S Afr Med J.

[22] Lehmann U, Dieleman M, Martineau T (2008). Staffing remote rural areas in middle-and low-income countries: a literature review of attraction and retention. BMC Health Serv Res.

